# Unveiling Endophytic Bacterial Community Structures of Different Rice Cultivars Grown in a Cadmium-Contaminated Paddy Field

**DOI:** 10.3389/fmicb.2021.756327

**Published:** 2021-11-16

**Authors:** Chaoqun Chu, Meiyu Fan, Chongyang Song, Ni Li, Chao Zhang, Shaowei Fu, Weiping Wang, Zhiwei Yang

**Affiliations:** ^1^College of Life Sciences, Capital Normal University, Beijing, China; ^2^State Key Laboratory of Hybrid Rice, Hunan Hybrid Rice Research Center, Changsha, China

**Keywords:** rice, endophytic bacteria, community structure, co-occurrence network, developmental stage, cadmium contamination, plant growth-promoting bacteria

## Abstract

Endophytic bacteria play potentially important roles in the processes of plant adaptation to the environment. Understanding the composition and dynamics of endophytic bacterial communities under heavy metal (HM) stress can reveal their impacts on host development and stress tolerance. In this study, we investigated root endophytic bacterial communities of different rice cultivars grown in a cadmium (Cd)-contaminated paddy field. These rice cultivars are classified into low (RBQ, 728B, and NX1B) and high (BB and S95B) levels of Cd-accumulating capacity. Our metagenomic analysis targeting 16S rRNA gene sequence data reveals that *Proteobacteria*, *Firmicutes*, *Actinobacteria*, *Acidobacteria*, *Bacteroidetes*, and *Spirochaetes* are predominant root endophytic bacterial phyla of the five rice cultivars that we studied. Principal coordinate analysis shows that the developmental stage of rice governs a larger source of variation in the bacterial communities compared to that of any specific rice cultivar or of the root Cd content. Endophytic bacterial communities during the reproductive stage of rice form a more highly interconnected network and exhibit higher operational taxonomic unit numbers, diversities, and abundance than those during the vegetative stage. Forty-five genera are significantly correlated with Cd content in rice root, notably including positive-correlating *Geobacter* and *Haliangium*; and negative-correlating *Pseudomonas* and *Streptacidiphilus*. Furthermore, Phylogenetic Investigation of Communities by Reconstruction of Unobserved States analysis shows that functional pathways, such as biosynthesis of siderophore and type II polyketide products, are significantly enhanced during the reproductive stage compared to those during the vegetative stage under Cd stress. The isolated endophytic bacteria from the Cd-contaminated rice roots display high Cd resistance and multiple traits that may promote plant growth, suggesting their potential application in alleviating HM stress on plants. This study describes in detail for the first time the assemblage of the bacterial endophytomes of rice roots under Cd stress and may provide insights into the interactions among endophytes, plants, and HM contamination.

## Introduction

Pollution of soil with heavy metal (HM) is widespread due to the rapid development of industry and extensive application of chemical fertilizers and pesticides (Huang and Jin, 2008; Atafar et al., 2010). HMs have harmful effects on crops and soil microorganisms and weaken the function of soil ecosystems (Kossoff et al., 2014; Guo et al., 2020). Therefore, remediation of HM-contaminated soil is a challenging task which holds great promise to improve the soil environment and to ensure safer food production.

Traditional methods for remediation of HM-contaminated soil rely heavily on physical and chemical approaches that are costly and non-sustainable. In recent years, biological remediation methods which take advantage of the metal-microbe interaction that occurs in plant hosts have been exploited (Etesami, 2018; Yin et al., 2019). Compared to bulk soil microbiomes, plant-associated microbiomes, such as endophytic and rhizospheric bacteria, have shown promise for HM remediation (Afzal et al., 2019). Endophytes are microorganisms living in the plant interior without causing disease. Beneficial endophytes not only promote plant growth but also help to remediate environmental contaminants (Etesami, 2018). Under metal stress, endophytic bacteria assist in phytoextraction and phytoremediation by increasing the biomass of HM-hyperaccumulating plants and enhancing the metal uptake by plants *via* acidifying the rhizosphere environment, thus enhancing the availability of metals (Rajkumar et al., 2012; Ma et al., 2016; Luo et al., 2019). In recent times, some HM-tolerant endophytes have been shown to promote plant growth in HM-contaminated soils and reduce the uptake of toxic metals by plants *via* biosorption, precipitation, complexation, and enzymatic transformation – ultimately leading to safer methods for human food production (Etesami, 2018; Yin et al., 2019).

Under HM stress, plants are capable of recruiting specific endophytic populations that enable a high HM resistance and plant growth-promoting properties (Croes et al., 2013; Pereira and Castro, 2014; Montalban et al., 2016; Syranidou et al., 2018; Sun et al., 2019; Zadel et al., 2020). Furthermore, these microbes have been shown to influence HM uptake by plants (Luo et al., 2017, 2019). For example, in the well-known cadmium (Cd)/zinc (Zn) superaccumulator *Sedum alfredii*, the enrichment of *Bacteroidetes* and depletion of *Firmicutes* and *Planctomycetes* in the endosphere of HP (hyperaccumulating) genotype may be responsible for the metal hyper-content in its shoots (Luo et al., 2017). Subsequent studies have shown that the abundance of *Streptomycetaceae*, *Nocardioidaceae*, and *Pseudonocardiaceae* is strongly correlated with an increased shoot biomass and total Cd/Zn accumulation in *S. alfredii* (Luo et al., 2019). An inoculation of *S. alfredii* with a synthetic bacterial community resulted in a significant improvement in plant biomass, root morphology, and Cd/Zn accumulation (Luo et al., 2019).

Compared to other crops, rice can easily incorporate more Cd and is the major source of dietary Cd intake in populations that rely principally on rice (Hu et al., 2016; Wang et al., 2019). Several studies have shown that bacteria inhabiting the rhizoplane and rhizosphere of rice can have a great influence on rice growth and uptake of HMs (Hu et al., 2015, 2019; Hou et al., 2018). However, few studies analyzing the endophytic bacteria under HM conditions in rice have been carried out. Zhou et al. (2020) showed that inoculation of rice endophytic *Stenotrophomonas* sp. R5-5 can change the relative abundance of some Cd-resistant bacteria in rice plants and downregulate the expression of rice Cd transporters, thereby reducing the accumulation of Cd in rice. An arsenic (As)-resistant facultative endophytic bacterium, *Serratia* sp. F2, can lower As accumulation in rice grain *via* increasing As adsorption by Fe plaque on the root surface (Cheng et al., 2020). These studies demonstrate that specific HM-tolerant microbes can significantly influence HM accumulation in rice. However, little is known about the composition and function of the microbiomes that colonize the endosphere of rice roots under HM stress. To better understand the ecological roles of endophytes with their host plants, it is crucial to investigate and characterize endophytic populations in rice grown in a HM-contaminated environment.

Here, we present a detailed investigation of the root bacterial endophytomes of rice grown in a Cd-contaminated paddy field using low- and high-Cd-accumulating cultivars. We employed bacterial 16S ribosomal RNA gene amplicon sequencing to (1) analyze the composition and dynamic changes of endophytic bacteria communities at two developmental stages of rice (2) decipher the keystone taxa that define the bacterial population structure (3) identify bacterial biomarkers that are uniquely associated with each individual rice cultivar, and (4) analyze the endophytic bacterial taxa correlated with Cd content. In addition, we identified metabolic pathways and functional genes enriched in root endophytic communities under Cd stress. Finally, we isolated and characterized bacterial endophytes from the Cd-contaminated rice roots as potential bio-inoculants to enhance crop productivity and mitigate HM stress.

## Materials and Methods

### Rice Seeds

Rice (*Oryza sativa* L.) seeds were provided by the Hunan Hybrid Rice Research Center (HHRRC), China. RBQ (Nipponbare), as a model plant, is a japonica cultivar. 728B, NX1B, BB, and S95B are indica cultivars bred by HHRRC. After years of cultivation, we observed that when soil-available Cd is 0.45–2.3mg/kg, RBQ, 728B, and NX1B accumulate 0.08–0.48mg Cd/kg grains, while BB and S95B accumulate 0.37–0.91mg Cd/kg grains. Because these two groups of cultivars accumulate significantly different amounts of Cd in grains, we classify RBQ, 728B, and NX1B as low-accumulating (LA) cultivars and BB and S95B as high-accumulating (HA) cultivars. The entire development phase for all five cultivars is 100 to 112days.

### Rice Cultivation and Sampling

From June to October 2018, five rice cultivars (RBQ, 728B, NX1B, BB, and S95B) were cultivated in a Cd-contaminated paddy field (27°58ʹN, 113°28ʹE) in Liling, Hunan province, China. This region is characterized by a humid subtropical monsoon climate with the following annual means: temperature, 18°C; sunshine, 1,500–1,910h; rainfall, 1,300–1,600mm; and frost-free period, 288days. The paddy field is contaminated by Cd due to a historic Zn-Pb ore mine in Liling. The soil of the experimental area has the following characteristics: pH 5.4; electrical conductivity, 198.7μs/cm; water content, 51.2%; organic content, 3.2%; organic carbon content, 13.7g/kg; total nitrogen content, 2.8g/kg; available phosphorus, 14.2mg/kg; available potassium, 181.9mg/kg; NH_4_^+^–N, 220.6mg/kg; and NO_3_^−^–N, 0.4mg/kg. Cd, chromium, manganese, copper, and lead are 0.9, 69.0, 53.4, 28.8, and 52.1mg/kg, respectively. Among them, Cd content is 0.9mg/kg, which greatly exceeds the risk screening value (0.3mg/kg) defined by China Risk for soil contamination on agricultural land (Trial; GB15618-2018).

The field experiment employed a randomized block design with five rice cultivars. Before use, the plot was fully ploughed and divided into 15 zones (200×60cm) with an interval of 40cm between each zone. There were three columns in one zone, 10 holes in one column, and two seedlings in each hole. There were three replicates (zones) for each cultivar. Non-polluted water was used for irrigation. Water and fertilizer management was carried out according to the local customs.

Thirty days (vegetative stage) and 60days (reproductive stage) after transplantation, rice root samples were collected. For each cultivar, five individuals were sampled according to diagonal sampling method in each zone, and the roots were mixed. The root samples were transported back to the laboratory under low temperature. Root samples were divided into two parts: one part was washed and air-dried, frozen in liquid nitrogen, and stored at −80°C for later DNA extraction and 16S rDNA amplification, while another part was stored at 4°C until isolation of endophytic bacteria. Root samples for the five rice cultivars at the vegetative stage were referred to as RBQ1, 728B1, NX1B1, BB1, and S95B1 and at the reproductive stage as RBQ2, 728B2, NX1B2, BB2, and S95B2.

### Determination of Cd Content in Roots

Roots were washed, deactivated at 105°C for 30min, and dried at 55°C for 48h and then 0.5g roots were pulverized in an agate mortar. The Cd content of root samples was determined using inductively coupled plasma mass spectrometry (ICP-MS) by Advanced Standards Technical Services Co., Ltd (Beijing). There were three replicates for each sample.

### DNA Extraction, PCR Amplification of 16S rDNA, and Sequencing

0.5g sample of rice roots from each cultivar was washed and surface-sterilized in triplicate, as described previously (Moronta-Barrios et al., 2018). There were three replicates for each cultivar. The effect of sterilization was verified by plating the last wash solution (100μl) on LB plates before proceeding with DNA extraction.

Endophytic bacterial DNA was extracted using the FastDNA^®^ Spin Kit for Soil (MP-Bio) according to the manufacturer’s protocols. DNA quantity and purity were measured using NanoDrop 2000 (Thermo Scientific, Wilmington, DE, United States). As described previously (Bulgarelli et al., 2015), the V5-V7 hypervariable regions of the bacterial 16S rRNA genes were amplified with primers 799F (5´-AACMGGATTAGATACCCKG-3´) and 1193R (5´-ACGTCATCCCCACCTTCC-3´) *via* thermocycler PCR system (GeneAmp 9700, ABI, United States). PCR reactions were conducted using the following settings: 3min initiation at 95°C, 27cycles of 30s denaturation at 95°C, 30s annealing at 55°C, and 45s elongation at 72°C, and a final extension at 72°C for 10min. PCR reactions were performed in triplicate with a 20μl mixture containing 4μl of 5× FastPfu Buffer, 2μl of 2.5mM dNTPs, 0.8μl of each primer (5μm), 0.4μl of FastPfu Polymerase, and 1μl (10ng/μl) of template DNA. Resulting PCR products were extracted from a 2% agarose gel and further purified using the AxyPrep DNA Gel Extraction Kit (Axygen Biosciences, Union City, CA, United States) before being quantified using the QuantiFluor^™^-ST (Promega, United States) according to the manufacturer’s protocol. Purified amplicons were pooled in equimolar amounts and paired-end sequenced (2×300bp) on an Illumina MiSeq platform (Illumina, San Diego, United States) according to the manufacturer’s protocols by Majorbio Bio-Pharm Technology Co. Ltd. (Shanghai, China).

### Processing of Sequencing Data

All raw Illumina Fastq files were demultiplexed and quality-filtered using Trimmomatic and merged using FLASH (Magoc and Salzberg, 2011). Operational taxonomic unit numbers (OTUs) were clustered with a 97% similarity cutoff using UPARSE (version 7.1),^1^ and chimeric sequences were identified and removed using UCHIME. The taxonomy of each 16S rRNA gene sequence was identified using the RDP Classifier algorithm^2^ against the Silva (SSU128) 16S rRNA database using a 70% confidence threshold.

### Bioinformatics and Statistical Analyses

Rarefaction curves, composition, and alpha diversity analyses were performed on https://cloud.majorbio.com/.Principal coordinates analysis (PCoA) was performed on weighted UniFrac distance matrices (Lozupone and Knight, 2005) using the “amplicon” package (Liu et al., 2021) in R (v3.6.1). Permutational multivariate analysis of variance (PERMANOVA) was conducted using the adonis function of Vegan in R. Co-occurrence network analyses were carried out using the “igraph” package (Csardi and Nepusz, 2006) in R, and networks were visualized using Gephi (v0.9.2; Bastian et al., 2009). Linear discriminant analysis effect size (LEfSe) was applied to the OTU table using Galaxy.^3^ Venn diagrams and Phylogenetic Investigation of Communities by Reconstruction of Unobserved States (PICRUSt) were constructed *via* ImageGP.^4^

SPSS v25.0 was used to perform paired sample *t*-tests, one-way ANOVA, and Spearman’s rank correlations. A heat map of the relative abundance of bacterial taxa or pathways was constructed in TBTools v1.089 (Chen et al., 2020).

### Isolation and Molecular Identification of Bacterial Isolates of Rice Roots

Endophytic bacteria of root were isolated as described by Zhou et al. (2020) with some modifications. 0.5g of the surface-sterilized roots was triturated aseptically in distilled water and inoculated into 30ml of R2A (Massa et al., 1998), TSB (tryptone 1.5%; soya peptone 0.5%; NaCl 0.5%; pH 7.4), or 1/2 LB broth (tryptone 0.5%; yeast extract 0.25%; NaCl 0.25%; pH 7.4), respectively. After incubation at 30°C for 24h, bacterial suspension was diluted appropriately. Aliquots of 100μl resuspension were spread on corresponding solid medium and incubated at 30°C until all colonies appeared. The colonies with distinctive morphological characteristics were picked and purified.

The isolates were identified by 16S rDNA sequencing. The gene was amplified using universal primers 27f (5´-AGAGTTTGATCCTGGCTCA-3´) and 1492r (5ʹ-GGTTACCTTGTTACGACTT-3´; Weisburg et al., 1991) and sequenced using an ABI 3730 Genetic Analyzer by RuiBiotech (Beijing). The sequences were used to perform BLASTN program against the 16S database of type strains at EzTaxon server (Kim and Chun, 2014).

### Characterization of Cd Tolerance and Plant Growth-Promoting Potential of Bacterial Isolates

We used the broth microdilution method to test the minimum inhibitory concentration (MIC) of Cd upon bacterial strains (Wiegand et al., 2008). A 96-well microtiter plate was taken from its sterile packing, and 50μl of each LB+CdCl_2_ dilution was added into the respective well (1st–10th), 100μl of LB broth in the control well (11th), and 50μl in the growth control well (12th). The bacterial suspension was adjusted to 1×10^8^ colony-forming unit/ml (equal to McFarland 0.5 standard) and then diluted 100 times. Each well containing the LB+CdCl_2_ solution (1st–10th) and the growth control well (12th) was inoculated with 50μl of the bacterial suspension, respectively. The final concentrations of CdCl_2_ in the 1st–10th wells were 5, 10, 20, 40, 80, 160, 320, 640, 1,280, and 2,560μm, respectively. The microtiter plate was incubated at 37°C for 16–20h. The MIC is defined as the lowest concentration of the antimicrobial agent that inhibits visible growth of the tested isolate as observed with the unaided eye. Each sample was repeated three times.

The PGP traits of bacterial isolates were quantitatively analyzed as follows: Indole-3-acetic acid (IAA) production was estimated using Salkowski reagent (Glickmann and Dessaux, 1995); siderophore production was determined using the Chrome Azurol S agar method (Schwyn and Neilands, 1987); phosphate solubilization estimations were carried out *via* the molybdenum blue method (Murphy and Riley, 1962), and 1-aminocyclopropane-1-carboxylate (ACC) deaminase activity was measured as described by Penrose and Glick (2003).

## Results

### Cd Accumulation in Roots of Five Cultivars at Two Stages

**Figure 1:**
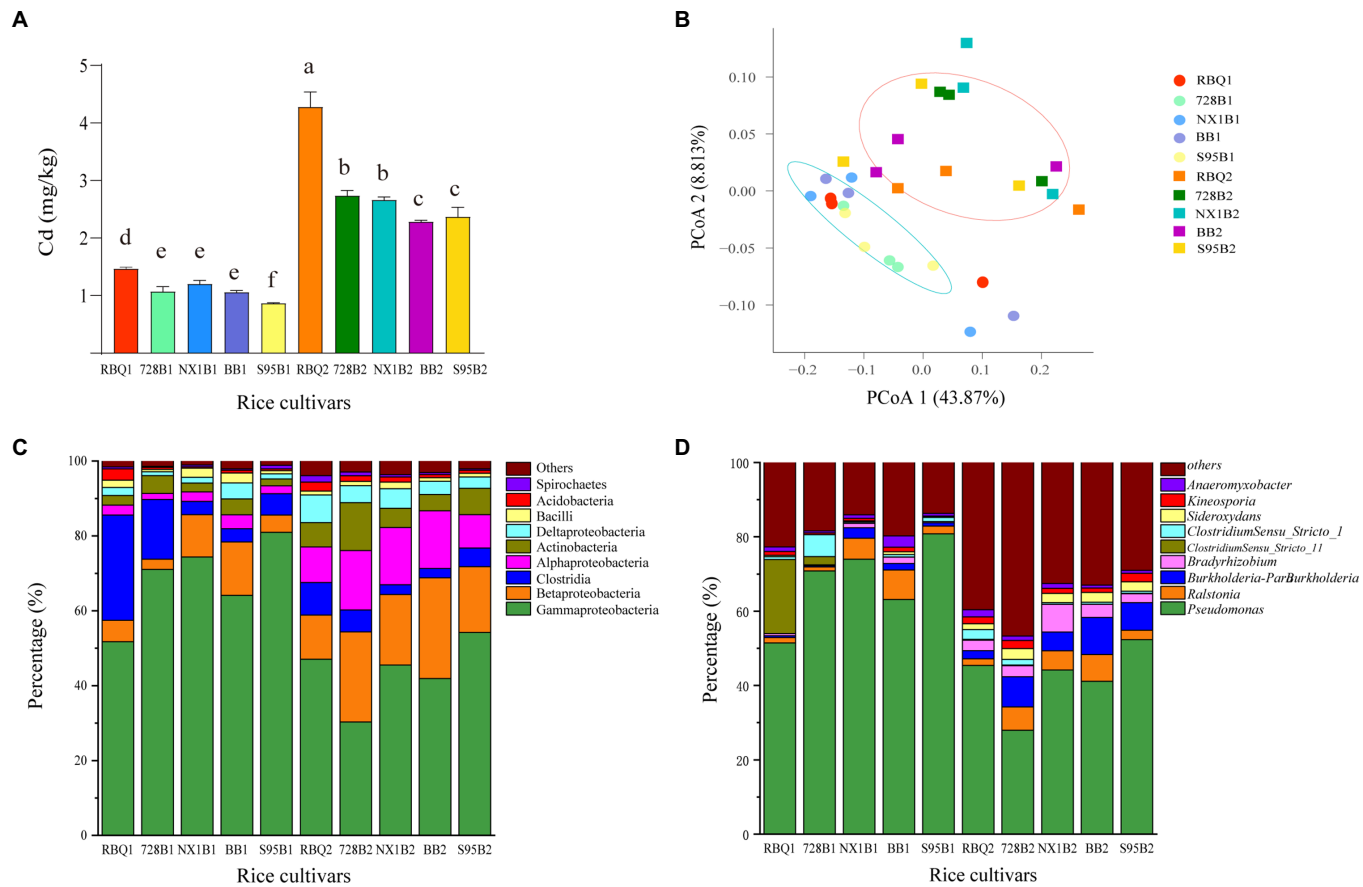
Cd content in roots of five rice cultivars at the vegetative and reproductive stages **(A)**; Principal coordinate analysis (PCoA) based on weighted UniFrac distance matrices at observed operational taxonomic unit (OTU) level **(B)**; relative abundances of the dominant classes **(C)** and genera **(D)**; hereafter, the cultivar name followed by 1 represents the vegetative stage and by 2 represents the reproductive stage. The different lowercase letters indicate significant differences among cultivars at *p*<0.05.

ICP-MS was used to determine Cd content in root samples collected at the vegetative and reproductive stage (Figure 1A). At the vegetative stage, RBQ accumulates more Cd than other cultivars. This phenomenon is more pronounced at the reproductive stage, when RBQ has a significantly higher Cd content, followed by LA cultivars (728B and NX1B) and HA cultivars (BB and S95B). It appears that the LA cultivars accumulate more Cd in roots than those of HA cultivars.

### Diversity and Abundance of Endophytic Bacterial Communities

A total of 1,236,157 16S rDNA sequences were obtained from root samples and were assigned to 2,261 OTUs based on 97% sequence similarity. Rarefaction curves of all samples tend to approach a saturation plateau, indicating that the sequencing accurately captures the endophytic communities of each rice cultivar (Supplementary Figure 1). The OTUs of rice cultivar RBQ, 728B, NX1B, BB, and S95B are 376, 309, 356, 448, and 359, respectively, at the vegetative stage and 686, 674, 691, 540, and 514, respectively, at the reproductive stage.

According to the taxonomic affiliations of the OTUs, there are 37 phyla (97.30% classified), 78 classes (92.30% classified), 175 orders (91.43% classified), 329 families (89.36% classified), and 614 genera (87.30% classified) in rice root endophytic communities. Among all rice cultivars, the dominant phyla include *Proteobacteria* (81.08%), *Firmicutes* (9.61%), *Actinobacteria* (5.16%), *Acidobacteria* (1.21%), *Bacteroidetes* (0.70%), and *Spirochaetes* (0.68%). The dominant classes include *Gammaproteobacteria* (56.13%), *Betaproteobacteria* (13.78%), *Clostridia* (8.15%), *Alphaproteobacteria* (7.76%), *Actinobacteria* (5.16%), *Deltaproteobacteria* (3.40%), *Bacilli* (1.43%), and *Acidobacteria* (1.21%; Figure 1C). The dominant genera include *Pseudomonas* (55.12%), *Ralstonia* (4.11%), *Burkholderia-Paraburkholderia* (3.90%), *Bradyrhizobium* (2.34%), *ClostridiumSensu_Stricto_1* (1.43%), *Sideroxydans* (1.37%), *Kineosporia* (1.43%), *Anaeromyxobacter* (1.23%), and *Bacillus* (1.10%; Figure 1D).

### Shifts of Bacterial Community Structures During Two Developmental Stage of Rice

PCoA was used to compare the bacterial community composition at the OTU level at two stages. PCoA1 and PCoA2 explain 43.87 and 8.81%, respectively, of the observed variation (Figure 1B). The samples from the vegetative stage and reproductive stage separate clearly into two groups, indicating that the developmental stage governs a larger source of variation. PERMANOVA based on weighted UniFrac distances confirms that the developmental stage significantly influences the composition of bacterial communities (14%, *p*<0.01). The rice cultivars do not differ significantly in bacterial compositions (*p*=0.25). The Cd content explains 2% of the observed variance, but not significantly different (*p*=0.493; Table 1).

**Table 1:** Permutational multivariate analysis of variance (PERMANOVA) results using weighted UniFrac distance matrices show effects of rice developmental stage, cultivar, and root Cd content on the bacterial community compositions.

Factor	% Explained	F. Model	*R* ^2^	value of *p*
Cultivar	15.07	1.32	0.15	0.25
Stage	14.04	4.90	0.14	0.011^*^
Cd	2.35	0.82	0.02	0.493
Cultivar: Stage	7.72	0.67	0.08	0.771
Cultivar: Cd	12.15	1.06	0.12	0.432
Stage: Cd	5.52	1.93	0.06	0.144
Cultivar: Stage: Cd	14.52	1.27	0.15	0.259
Residuals	28.64		0.29	
Total	100.00		1.00	

* denotes significant differences at p<0.05.

**Figure 2:**
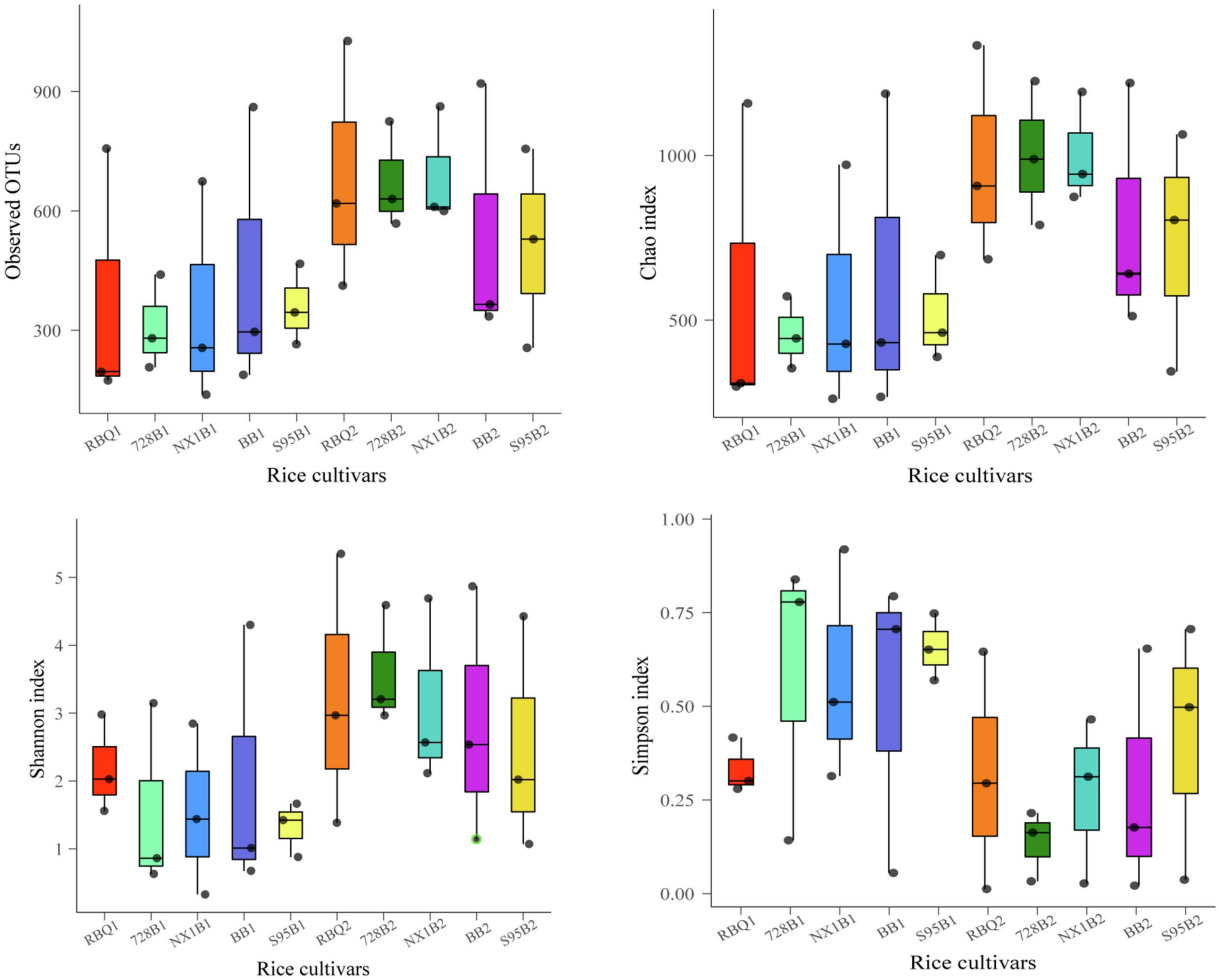
Box plots of α-diversity indices show observed OTUs, Shannon index, ACE index, and Chao1 of five rice cultivars at the vegetative and reproductive stages. The lines at the top, bottom, and middle of the box correspond to the 75th, 25th, and 50th percentiles (median), respectively. The ends of whiskers correspond to the minimum and maximum value.

In order to demonstrate the composition shifts of the endophytic bacterial communities at two developmental stages, two-sample *t*-tests were used to compare α-diversity indices. The results shows that observed OTUs, Shannon index, ACE index, and Chao1 at the reproductive stage are significantly higher than those at the vegetative stage (*p*<0.01), while the Simpson index at the reproductive stage is significantly lower than that at the vegetative stage (*p*<0.01), indicating that the endophytic bacterial community has much higher richness and diversity at the reproductive stage compared to the vegetative stage (Supplementary Table 1). ANOVA shows that α-diversity indices among different cultivars are not statistically different (Figure 2, Supplementary Table 2).

The relative abundances of dominant endophytic bacterial taxa were further compared between two developmental stages. In comparison with the vegetative stage, the relative abundance of *Alphaproteobacteria*, *Betaproteobacteria*, *Deltaproteobacteria*, and *Actinomycetes* at the reproductive stage are significantly higher (*p*<0.05), whereas the relative abundance of *Gammaproteobacteria* is significantly lower (*p*<0.01). The relative abundance of *Clostridia*, *Bacilli*, and *Acidobacteria* does not show significant changes (Supplementary Table 3). At the genus level, the relative abundance of *Burkholderia-Paraburkholderia*, *Bradyrhizobium*, *Sideroxydans*, and *Kineosporia* is significantly higher (*p*<0.01), whereas the abundance of *Pseudomonas* is significantly lower (*p*<0.01). The relative abundance of *Ralstonia*, *Clostridium_Sensu_Stricto_1*, *Clostridium_Sensu_Stricto_11*, *Anaeromyxobacter*, and *Bacillus* does not show significant changes (Supplementary Table 4).

### Co-occurrence Network Analysis of the Bacterial Community

**Figure 3:**
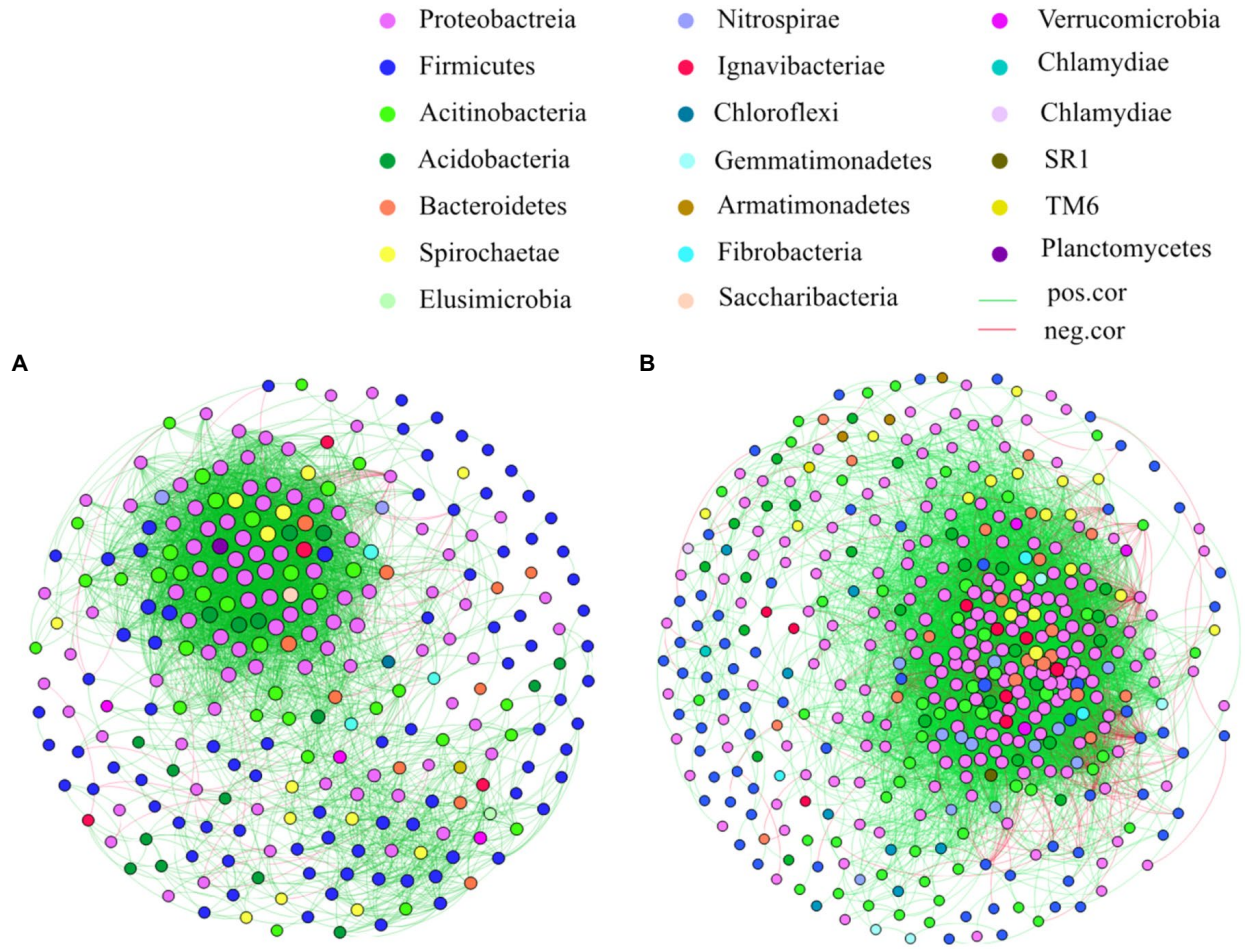
Co-occurrence network diagrams of samples at the vegetative **(A)** and reproductive **(B)** stages. Connections stand for Spearman’s correlation with *r*>0.75 and statistical significance at *p*<0.01. Nodes are colored by taxonomy, and the size of each node is proportional to the number of connections (i.e., degree). Green and red lines represent co-occurrence and mutual exclusion, respectively. Details regarding the co-occurrence network are reported in Supplementary Tables 5, 6.

Co-occurrence bacterial network analysis shows different connectivity patterns at the two developmental stages. In general, the number of correlations is substantially larger at the reproductive stage than at the vegetative stage. At the vegetative stage, the network has 266 nodes with 4,277 edges, among which 4,215 are positively correlated and 62 are negatively correlated, and the average degree of the network is 32.16. At the reproductive stage, the network has 425 nodes with 11,659 edges, among which 10,763 are positively correlated, and 896 are negatively correlated, and the average degree of the network is 54.87 (Figure 3, Supplementary Table 5). These findings indicate a higher complexity of the bacterial networks at the reproductive stage than at the vegetative stage.

The taxonomy of the most connected nodes varies between the vegetative and reproductive stages. At the vegetative stage, *Actinobacteria* accounts for 17.36% of the total degree of connection, followed by *Betaproteobacteria* (16.79%), *Deltaproteobacteria* (14.37%), *Alphaproteobacteria* (11.34%), and *Clostridia* (9.84%). At the reproductive stage, *Betaproteobacteria* accounts for 23.89% of the total degree of connection, followed by *Deltaproteobacteria* (22.35%), *Actinobacteria* (9.16%), *Alphaproteobacteria* (7.15%), and *Acidobacteria* (6.38%; Supplementary Table 6).

According to degree centrality, we classified the most central 20% as key nodes (Xian et al., 2020). The relative abundance of the key nodes at each developmental stage varies greatly, ranging from 0.005% to 1%. Several of the highly connected taxa, such as *Anaeromyxobacter*, *Geobacter*, *Haliangium*, *kineosporia*, and *Streptomyces* at the vegetative stage and *Ralstonia*, *Haliangium*, *Sideroxydans*, *Geobacter*, *Anaeromyxobacter, Bradyrhizobium* and *Micromonospora* at the reproductive stage, appear to be the keystone bacterial taxa (Supplementary Figure 2).

### Cultivar-Associated Bacterial Biomarkers and Specific OTUs

**Figure 4:**
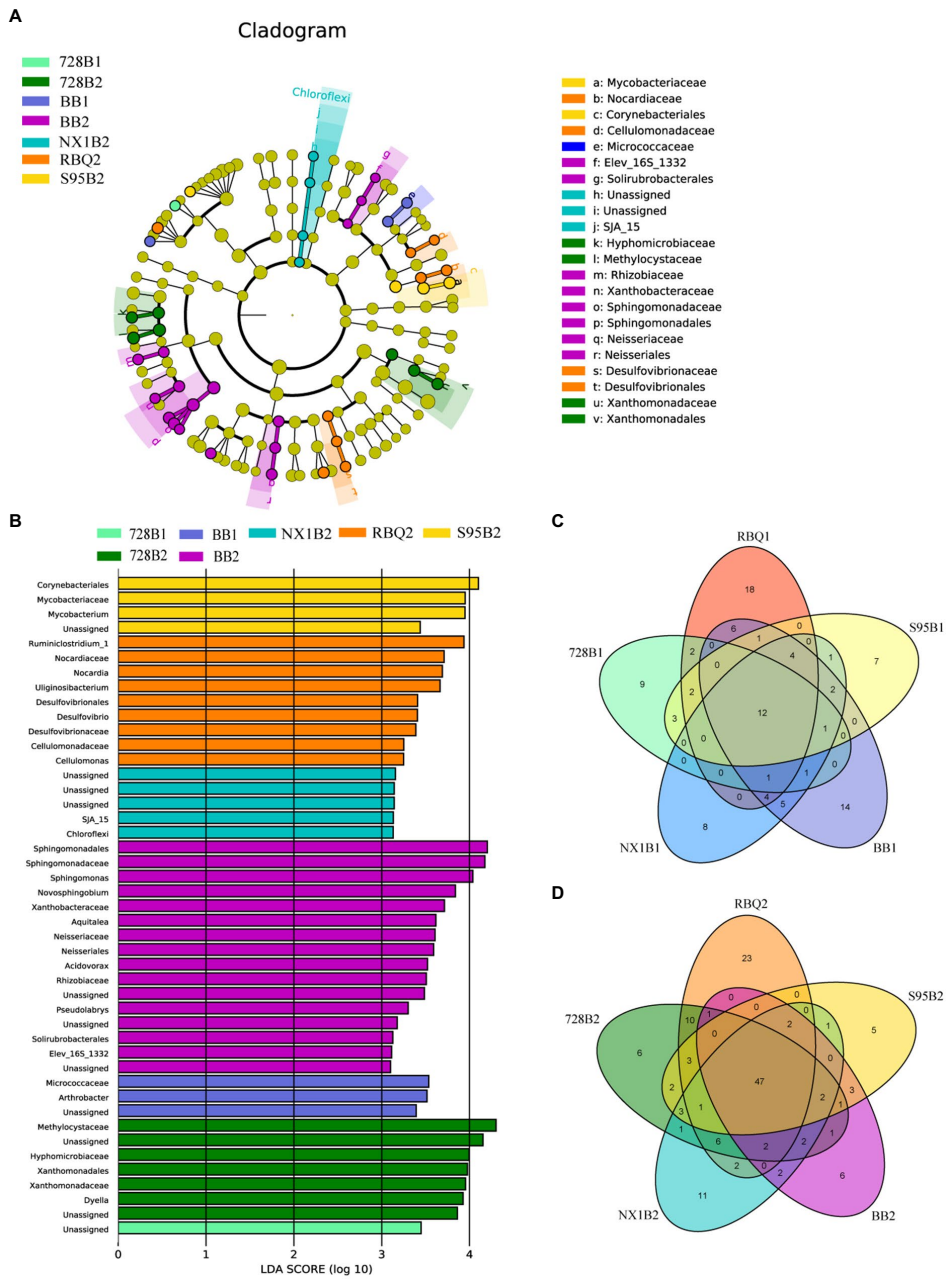
**(A–B)**Taxonomic cladograms of linear discriminant analysis effect size (LEfSe) analysis of rice cultivars at two developmental stages, depicting OTUs with absolute linear discriminant analysis (LDA) scores larger than 3. Venn diagrams show shared and specific OTUs among five rice cultivars at the vegetative **(C)** and reproductive **(D)** stages.

In order to detect the bacterial taxonomic biomarkers of each cultivar, OTUs with relative abundance greater than 0.05% were compared between samples using LEfSe [linear discriminant analysis (LDA) effect size]. OTUs with the highest LDA (Wilcoxon *p*<0.05, LDA score>3) from each cultivar are shown in Figure 4. At the vegetative stage, only cultivar 728B and BB have differentially abundant clades, such as *Micrococcaceae* and *Arthrobacter* in BB. However, at the reproductive stage, all rice cultivars show differentially abundant clades, such as *Ruminiclostridium_1* and *Nocardia* in RBQ; *Methylcystiaceae* and *Hyphomicrobiaceae* in 728B; *Chloroflexi* in NX1B; *Sphingomonadaceae* and *Sphingomonas* in BB; and *Mycobacteriaceae* and *Mycobacterium* in S95B (Figures 4A,B).

The Venn diagram shows a cluster of shared OTUs and the specific OTUs for each cultivar. At the vegetative stage, there are 12 shared OTUs (77.8%) among different cultivars, mainly including *Pseudomonas* (OTU_538, 68.01%), *Ralstonia* (OTU_1351, 3.64%), and *Clostridium_Sensu_Stricto_1* (OTU_83, 1.53%). Specific OTUs of RBQ, 728B, NX1B, BB, and S95B at the vegetative stage account for 7.71, 5.66, 1.09, 2.81, and 1.19% of the bacterial communities, respectively (Figure 4C).

At the reproductive stage, there are 47 shared OTUs (77.60%) among different cultivars, mainly including *Pseudomonas* (OTU_538, 42.17%), *Ralstonia* (OTU_1351, 4.52%), *Burkholderia-Paraburkholderia* (OTU_1265, 3.31%; OTU_1568, 2.77%), *Sideroxydans* (OTU_2233, 1.88%), and *Bradyrhizobium* (OTU_1319, 1.88%; OTU_389, 1.71%). Specific OTUs of RBQ, 728B, NX1B, BB, and S95B at the reproductive stage account for 3.64, 7.62, 1.99, 1.33, and 1.05% of the bacterial communities, respectively (Figure 4D).

### Bacterial Taxa Correlated With Cd Contamination

**Figure 5:**
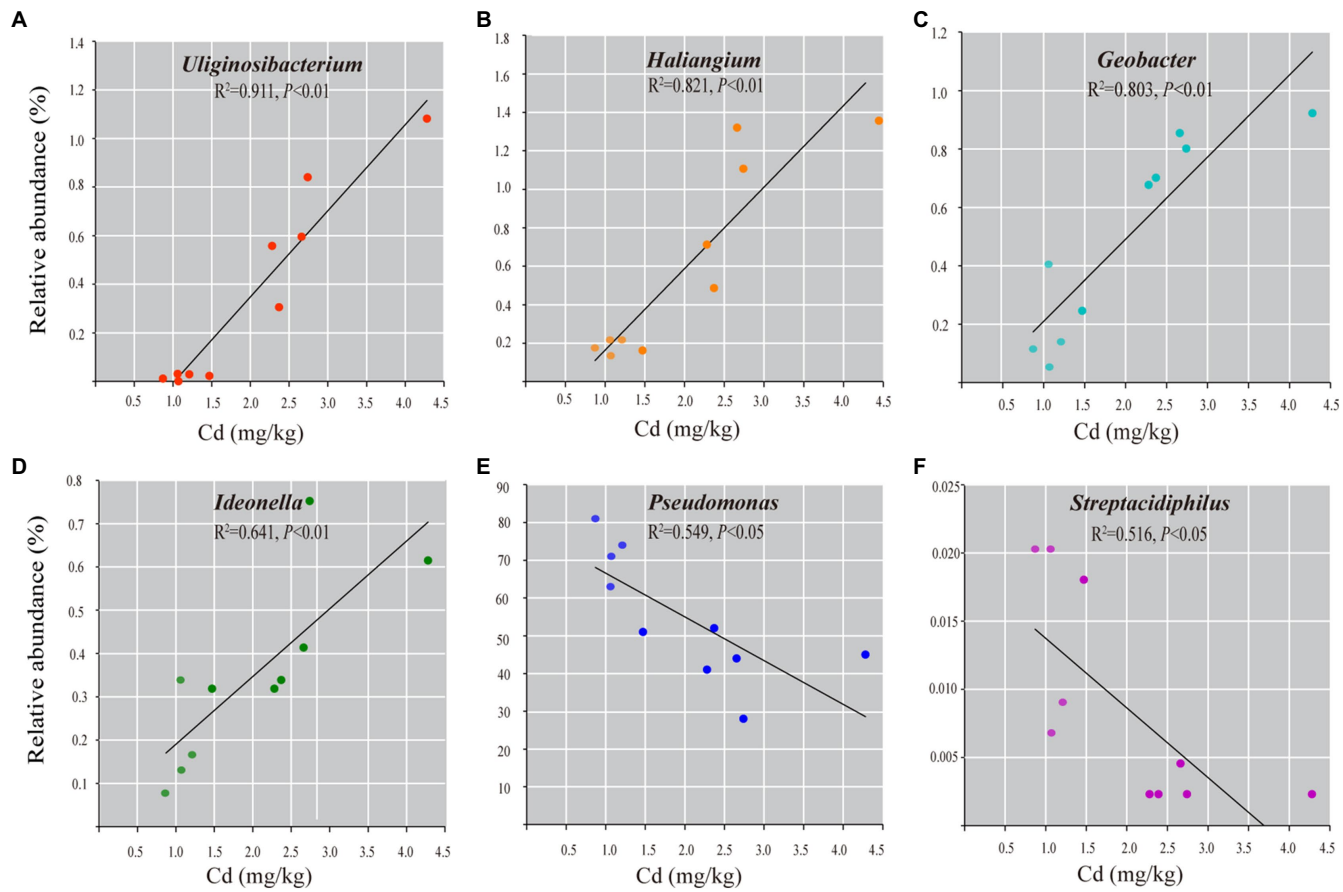
The correlation analysis of the representative bacterial genera with Cd content in roots of rice grown in Cd-contaminated soil. Linear regression analyses show the relationships between the relative abundance of the genera of *Geobacter* **(A)**, *Uliginosibacterium* **(B)**, *Haliangium* **(C)**, *Ideonella* **(D)**, *Pseudomonas* **(E)**, and *Streptacidiphilus* **(F)** and Cd content (mg/kg dry weight). Only statistically significant correlations at *p*<0.01 are shown.

Spearman correlation analysis shows that 45 genera (7.3% of observed genera) are significantly correlated with Cd content (Spearman’s *r*>0.75, *p*<0.01, Supplementary Table 7). Among them, forty-one genera are positively correlated with Cd content, such as *Geobacter*, *Haliangium*, *Uliginosibacterium*, *Ideonella*, *Micromonospora*, and *Geothrix*. Four genera are negatively correlated with Cd content, including *Pseudomonas*, *Arthrobacter*, *Streptacidiphilus*, and *Prolixibacter*. Linear regressions between the abundances of the representative bacterial genera and the Cd content confirm the abundance of unique bacteria taxa correlated with the content of Cd in roots (Figure 5).

### Prediction of Functional Profiles of the Bacterial Community

**Figure 6:**
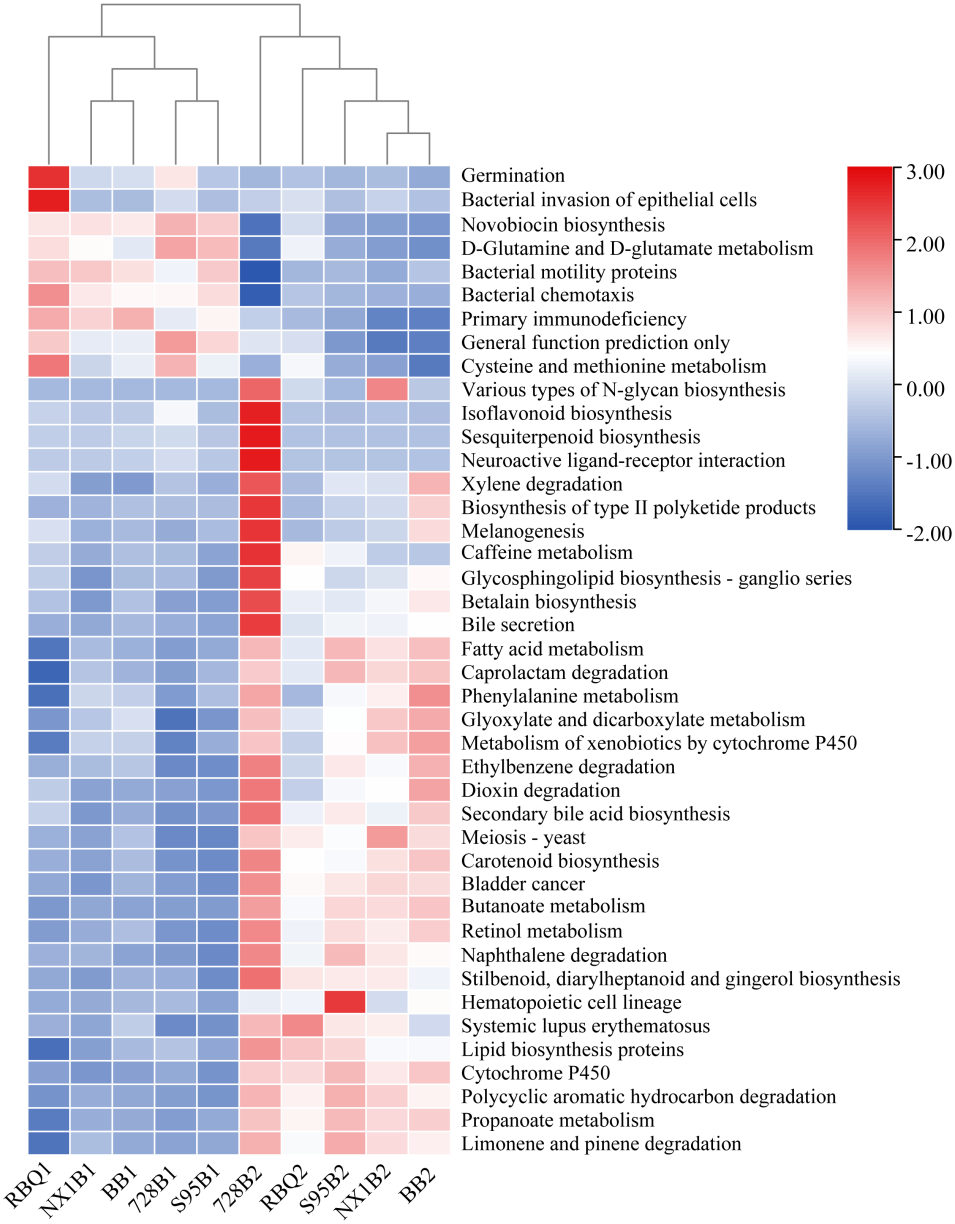
Predicted gene functional categories of the endophytic microbiome of five rice cultivars at the vegetative and reproductive stages. The heat map is drawn for a total of 42 metabolic pathways which were significantly changed from the vegetative to reproductive stage.

A total of 297 functional pathways were analyzed for endophytic bacterial communities. KEGG Orthology (KO)-based PcoA analysis reveals functional differences of five rice cultivars across two stages (Supplementary Figure 3). From the vegetative to the reproductive stage, a total of 42 metabolic pathways (representing 14% of predicted functions) are significantly changed (*p*<0.05), including bacterial motility proteins, butanoate metabolism, propanoate metabolism, bacterial chemotaxis, lipid biosynthesis proteins, fatty acid metabolism, glyoxylate and dicarboxylate metabolism, cysteine and methionine metabolism, and the biosynthesis of type II polyketide products (Figure 6).

Grime’s Competitor/Stress-tolerator/Ruderal (CSR) theory proposes that organisms in a community face a three-way resource trade-off: competition with neighbors for resources (Competitive traits); survival in underproductive environments (Stress-tolerant traits); and survival in highly disturbed environments (Ruderal traits; Grime, 1977; Grime and Pierce, 2012). According to CSR theory and the detailed criteria for functional traits described in Wood et al. (2018), six functional pathways are classified as C traits, 11 as S traits, and seven as R traits in this study (Supplementary Table 8). Comparison of CSR traits between the two developmental stages reveals that the relative abundances of C traits (biosynthesis of siderophore and type II polyketide products) and S traits (Porphyrin and chlorophyll metabolism and Proteasome) are significantly increased at the reproductive stage. Most metabolic pathways in the R traits are also enhanced, especially carbon fixation pathways, citrate cycle (TCA cycle), and oxidative phosphorylation.

### Cd Tolerance and PGP Potential of Cultivable Bacteria

Forty-three strains isolated from Cd-contaminated rice roots are ascribed to 12 genera in five classes (Supplementary Table 9). Among them, *Bacillus* comprises the largest number of taxa (53.5%), followed by *Klebsiella* (11.6%), *Paenibacillus* (7%), and *Herbaspirillum* (7%).

**Figure 7:**
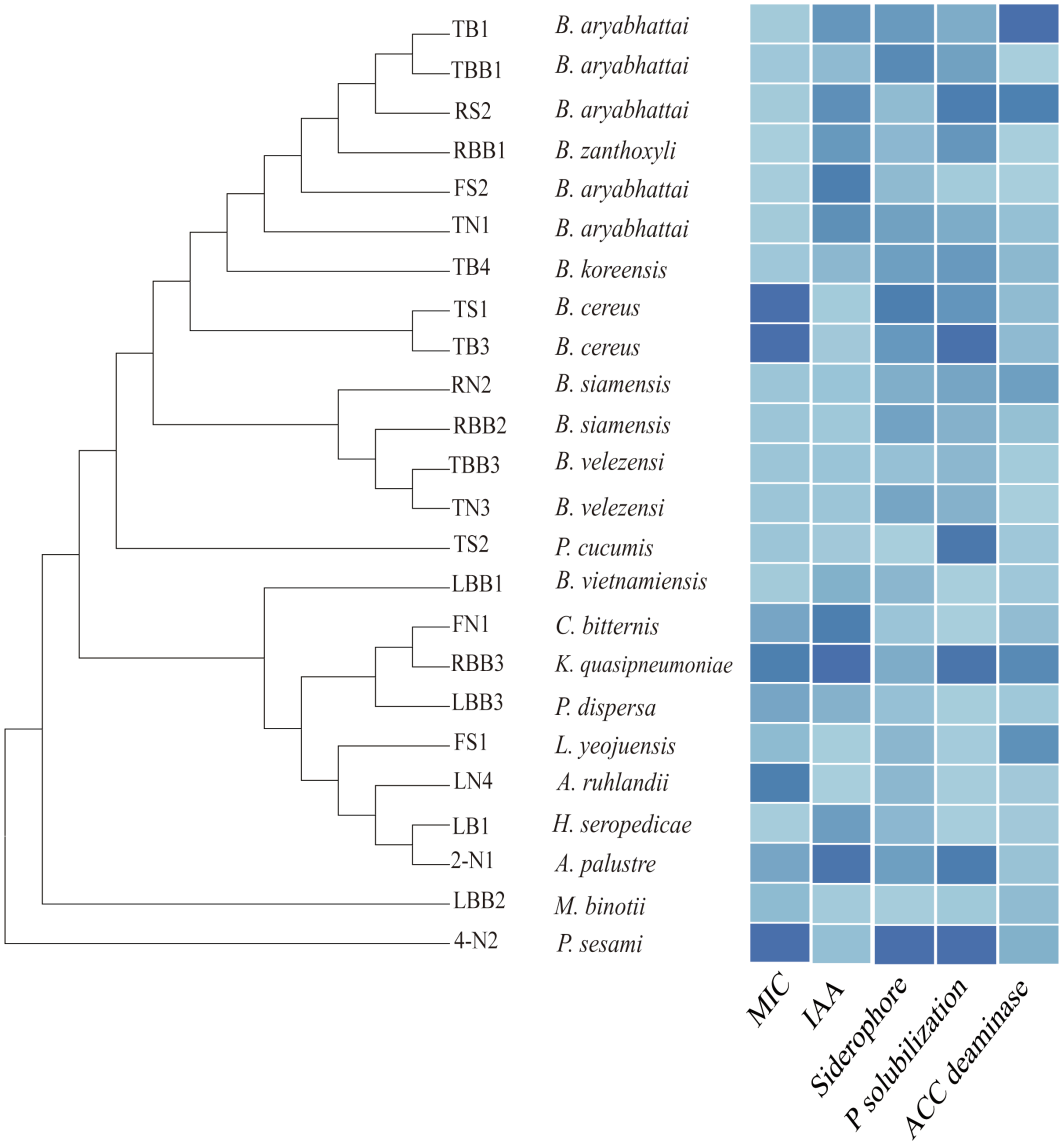
Phylogenetic tree of the representative bacterial isolates and their minimum inhibitory concentrations (MICs) to Cd and plant growth-promoting (PGP) traits, including production of indole-3-acetic acid (IAA), siderophore, phosphate solubilization, and 1-aminocyclopropane-1-carboxylate (ACC) deaminase activities. The color gradient from dark to light represents the value from high to low.

We tested the Cd tolerance and PGP traits of some representative isolates (Figure 7, Supplementary Table 10). The minimum inhibitory concentrations (MICs) of bacterial isolates to Cd are between 10 and 1,280μm, among which *Bacillus* sp. TB3 and TS1 show the highest tolerance (1,280μm). As for PGP traits, *Klebsiella* sp. RBB3 shows the highest IAA production (52.84μg/ml). *Pseudomonas* sp. 4-N2 shows the highest siderophore production (96.85mg/l) and phosphate solubilization ability (41.46μg/ml). The activity of ACC deaminase is highest for *Bacillus* sp. TB1 (12.84U/mg), whereas some isolates are negative for the assay. Most of the endophytic bacteria isolated from the Cd-contaminated roots show traits of a higher Cd resistance and PGP potential. The endophytic bacteria with those traits can be exploited as potential bioinoculants to enhance crop productivity and mitigate HM stress.

## Discussion

High-throughput sequencing studies have revealed a high diversity of endophytic bacteria in rice roots (Sun et al., 2008; Edwards et al., 2015; Ding et al., 2019; Walitang et al., 2019). In this study, by using five cultivars grown in a natural Cd-contaminated paddy field, we observed *Proteobacteria*, *Firmicutes*, *Actinobacteria*, *Acidobacteria*, *Bacteroidetes*, and *Spirochaetes* are the most dominant phyla, and *Pseudomonas*, *Ralstonia*, *Burkholderia-Paraburkholderia*, *Bradyrhizobium*, *ClostridiumSensu_Stricto_1*, *Sideroxydans*, *Kineosporia*, *Anaeromyxobacter*, and *Bacillus* are the most dominant genera.

The endophytic microbiota has been found to be beneficial to plant growth, nutrition uptake, and resistance to biotic and abiotic stresses (Castrillo et al., 2017; Kim and Lee, 2020). In this study, we found that *Proteobacteria* makes up the largest fraction of the bacterial communities, which include gamma, alpha, beta, and delta subclasses. In *Alphaproteobacteria*, *Bradyrhizobium* and *Sphingomonas* are two representatives reported to be nitrogen fixers (Knief et al., 2012; Sessitsch et al., 2012; Hardoim et al., 2015). In *Betaproteobacteria*, *Burkholderia-Paraburkholderia* shows multiple phytobeneficial traits (Madhaiyan et al., 2008; King et al., 2019). *Pseudomonas*, affiliated to the *Gammaproteobacteria*, is the most abundant genus, which has been detected in various rice tissues, including root, stem, leaves, and seeds (Walitang et al., 2019). It has been reported that *Pseudomonas* can synthesize PGP substance (such as phytohormone) to facilitate plant growth or produce antibiotics to increase resistance to pathogens (Weller et al., 2002; Sivasakthi et al., 2013; Han et al., 2015). We found that *Deltaproteobacteria* is another dominant bacterial population in rice root. Compared with the root-associated bacteria of other crops, rice root is significantly enriched for *Deltaproteobacteria* (Ding et al., 2019). One possible explanation is that the flooded nature of rice cultivation could favor the growth of anaerobic microorganisms in the *DeltaProteobacteria*, such as *Geobacter* and *Anaeromyxobacter*, which are involved in the reduction of Fe (III) and S (Ding et al., 2015; Sun et al., 2015).

Endophytic microbiota change with the growth and developmental stages of host plants (Santoyo et al., 2016). In this study, the alpha diversity indices (Observed OTUs, Shannon, ACE and Chao 1) of bacterial communities at the reproductive stage are significantly higher than that at the vegetative stage, indicating a more mature community is established at the reproductive stage. Other studies also reported that rice root microbiota varies dramatically during the vegetative stage and becomes stabilized during the reproductive stage until rice ripening (Edwards et al., 2018; Zhang et al., 2018). Xun et al. (2021) found that bacterial communities with a higher phylogenetic diversity are more stable and more resistant to environmental disturbance. Our co-occurrence network analysis further reveals that the endophytic bacterial community has established a complex symbiotic relationship during the reproductive stage. It is worth noting that although *Pseudomonas* and *Burkholderia-Paraburkholderia* are the most dominant genera, they are not in the top 20% key nodes of the network. In contrast, *Sideroxydans*, *Ralstonia*, *Haliangium*, *Bradyrhizobium*, *Micromonospora*, *Anaeromyxobacter*, and *Geobacter* are the key nodes of the network. Among them, some species have been reported to participate in nitrogen fixation or CO_2_ fixation (Knief et al., 2012; Federico et al., 2015), phosphate solubilizing (Carro et al., 2018), and the iron cycle (Weiss et al., 2007; Ding et al., 2015). We suggest that the fundamental metabolic functions (especially nitrogen and carbon metabolism, phosphorus metabolism, and iron cycle) are the key engines for the assemblage of rice endophytic bacterial communities, which is in agreement with what has been found in soil microbiome (Xun et al., 2021).

Several studies have demonstrated that HM hyper-accumulating or tolerant plants harbor endophytic populations with a high HM resistance, indicating that an environmental stress influences the endophytic microbiomes (Luo et al., 2019; Zadel et al., 2020). As the Cd content in roots is significantly different among LA and HA cultivars, we further analyzed whether specific bacterial taxa may be affected by Cd contamination. Spearman correlation analysis shows that 45 genera are significantly correlated with Cd content. Some genera, which are positively correlated to Cd content, were also reported in other HM-polluted environments. For example, *Geobacter* and *Geothrix* are the two dominant genera in the uranium mine sediment (Li et al., 2018). Recent studies revealed that *Geobacter*, as a Fe (III)-reducing bacterium, plays an important role in controlling the mobility of As in the rice rhizosphere (Dai et al., 2020; Li et al., 2021). The negatively correlated genera, such as *Pseudomonas* and *Arthrobacter*, have been reported as the most represented genera in HM-contaminated sites (Pires et al., 2017). The inoculation of seeds with *Pseudomonas* or *Arthrobacter* has been shown to facilitate the root development of *Brassica napus* L. under the Zn/Cd stress (Croes et al., 2013; Montalban et al., 2016). In this study, *Pseudomonas* and *Arthrobacter* are enriched in roots with a low Cd content, suggesting their potential applications in alleviating HM stress in host plants.

In order to understand the characteristics of metabolic pathways of rice root endophyte under HM stress, PICRUSt and CSR theory (Grime, 1977; Grime and Pierce, 2012) were used to conduct a preliminary analysis of the functional characteristics of the endophytic bacterial communities. Wood et al. (2018) reveal that a bacterium’s competitive attributes are critical for its ability to occupy and proliferate in a Cd-contaminated rhizosphere. In this study, we observe that the bacterial biosynthesis of type II polyketide products and siderophore (C traits) is enriched at the reproductive stage, indicating that endophytic bacteria might promote host plant resistance to pathogens by synthesizing polyketide antibiotics and contribute to Fe uptake by siderophore production. Siderophores may also participate in chelating HM ions and thus reduce the toxicity of Cd to host plants (Etesami, 2018). Among S traits, porphyrin metabolism and proteasome function increase significantly. Mn-porphyrins are potent antioxidants, scavenging O^2−^ radicals (Miriyala et al., 2012), and proteasomes are involved in protein degradation and the recycling of amino acids (Becker and Darwin, 2017), suggesting that these two pathways may be involved in alleviating HM-induced oxidative stress and removal of damaged proteins. As for R traits, the enhancement of carbon fixation pathways, citrate cycle (TCA cycle), and oxidative phosphorylation is favorable for endophytes to utilize nutrients for proliferation and occupying ecological niches.

From the above studies based on 16S rRNA gene amplicon sequencing and bioinformatic analysis, we profiled the root endophytic bacterial populations under Cd contamination. Based on these studies, we decided to isolate beneficial endophytic strains with potential applications in HM remediation. Plant growth-promoting bacteria (PGPB) are known to play a vital role in enhancing crop productivity and plant resistance to HM stresses (Etesami, 2018; Guo et al., 2020). Some genera affiliated to *Bacillus*, *Neorhizobium*, *Delftia*, *Pseudomonas*, *Cupriavidus*, and *Stenotrophomonas* have been reported to enhance rice growth and reduce the accumulation of HM in grains (Siripornadulsil and Siripornadulsil, 2013; Lin et al., 2016; Li et al., 2017; Liu et al., 2018; Treesubsuntorn et al., 2018). In this study, we isolated cadmium-resistant endophytic bacteria from 12 genera, including *Bacillus*, *Paenibacillus*, *Klebsiella*, *Herbaspirillum*, and *Pseudomonas*. Most of them show multiple PGP activities, such as phosphate solubilization, production of phytohormones (e.g., IAA), siderophores, and ACC deaminase. These activities suggest their beneficial roles in improving rice growth under stress conditions. Further studies are necessary to understand the highly complex interactions between rice and its associated microbe and to exploit the endophytic PGPB for remediation of HM contamination in paddy fields.

## Data Availability Statement

The datasets presented in this study can be found in online repositories. The names of the repository/repositories and accession number(s) can be found at: https://www.ncbi.nlm.nih.gov/, SRR11528738-SRR11528760 and MZ923694-MZ923717.

## Author Contributions

MF, NL, and CZ carried out the samples preparation and bacterial gDNA extraction. CC, MF, and CS performed bioinformatics and statistics analyses. CC and SF analyzed the Cd resistance and PGP traits of the isolates. CC and MF wrote the manuscript draft under the supervision of WW and ZY. All authors contributed to the article and approved the submitted version.

## Funding

This research was financially supported by the Agricultural Science-Technology Innovation Fund of Hunan Province, China (grant no. 2018QN03) and the Science and Technology Program of Changsha, China (grant no. kq1801033).

## Conflict of Interest

The authors declare that the research was conducted in the absence of any commercial or financial relationships that could be construed as a potential conflict of interest.

## Publisher’s Note

All claims expressed in this article are solely those of the authors and do not necessarily represent those of their affiliated organizations, or those of the publisher, the editors and the reviewers. Any product that may be evaluated in this article, or claim that may be made by its manufacturer, is not guaranteed or endorsed by the publisher.

## Footnotes

^1^ http://drive5.com/uparse/

^2^ http://rdp.cme.msu.edu /

^3^ http://huttenhower.sph.harvard.edu/galaxy/

^4^ http://www.ehbio.com/ImageGP/index.php/Home/Index/index.html
