# Supplementary material for: Unveiling Endophytic Bacterial Community Structures of Different Rice Cultivars Grown in a Cadmium-Contaminated Paddy Field
Source: Front Microbiol. 2021 Nov 16;12:756327. doi: 10.3389/fmicb.2021.756327 (PMC8635021; doi:10.3389/fmicb.2021.756327)
Supplement: Supplementary file 1 [file Data_Sheet_1.PDF]

Supplementary material

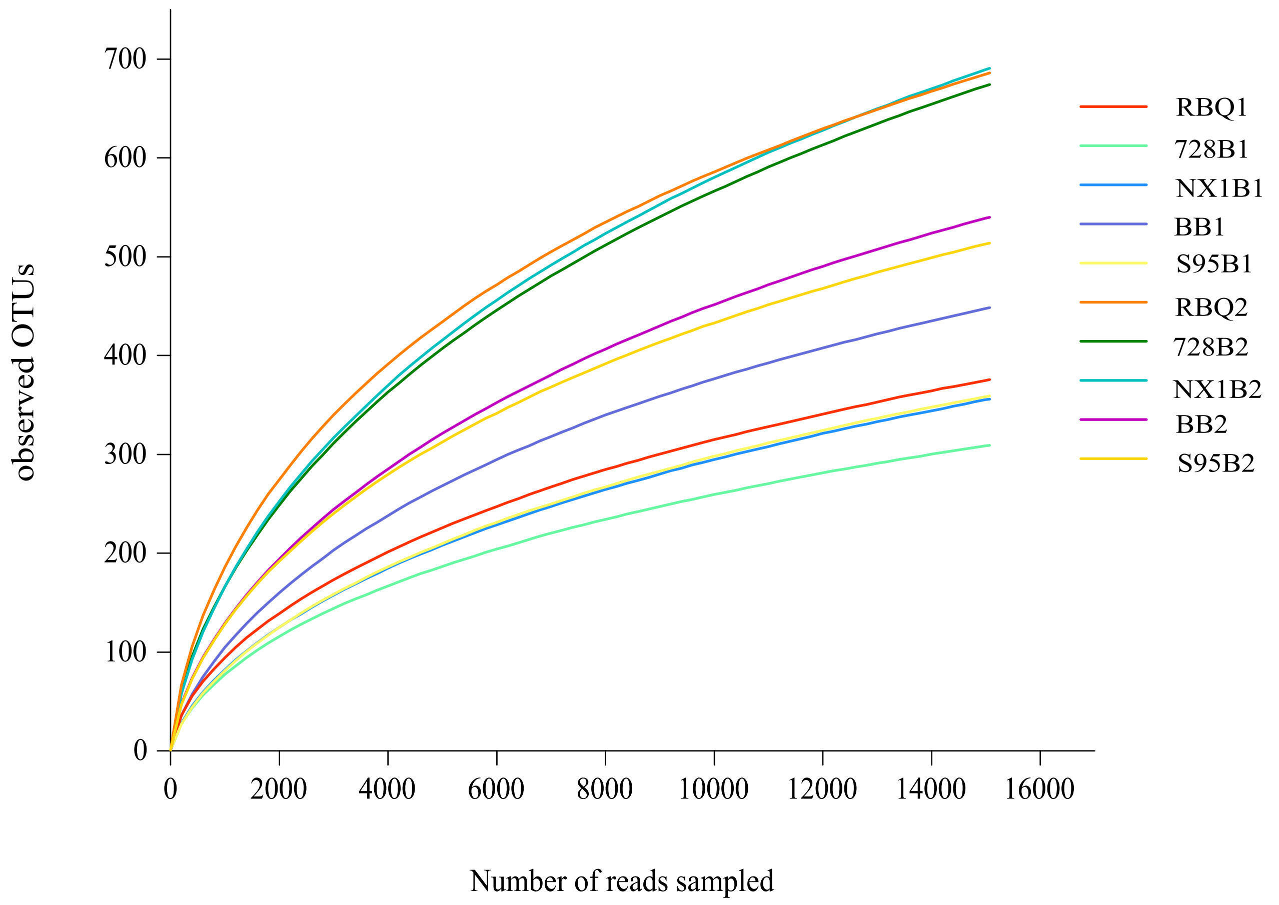

Supplementary Figure 1 Dilution curves of observed operational taxonomic units (OTUs) from 5 rice cultivars at the vegetative and reproductive stages
