# Supplementary figures and images for: Unveiling Endophytic Bacterial Community Structures of Different Rice Cultivars Grown in a Cadmium-Contaminated Paddy Field
Source: Front Microbiol. 2021 Nov 16;12:756327. doi: 10.3389/fmicb.2021.756327 (PMC8635021; doi:10.3389/fmicb.2021.756327)

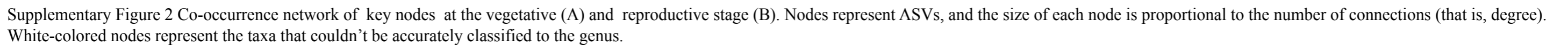

Supplement: Supplementary file 2 [file Data_Sheet_2.PDF]

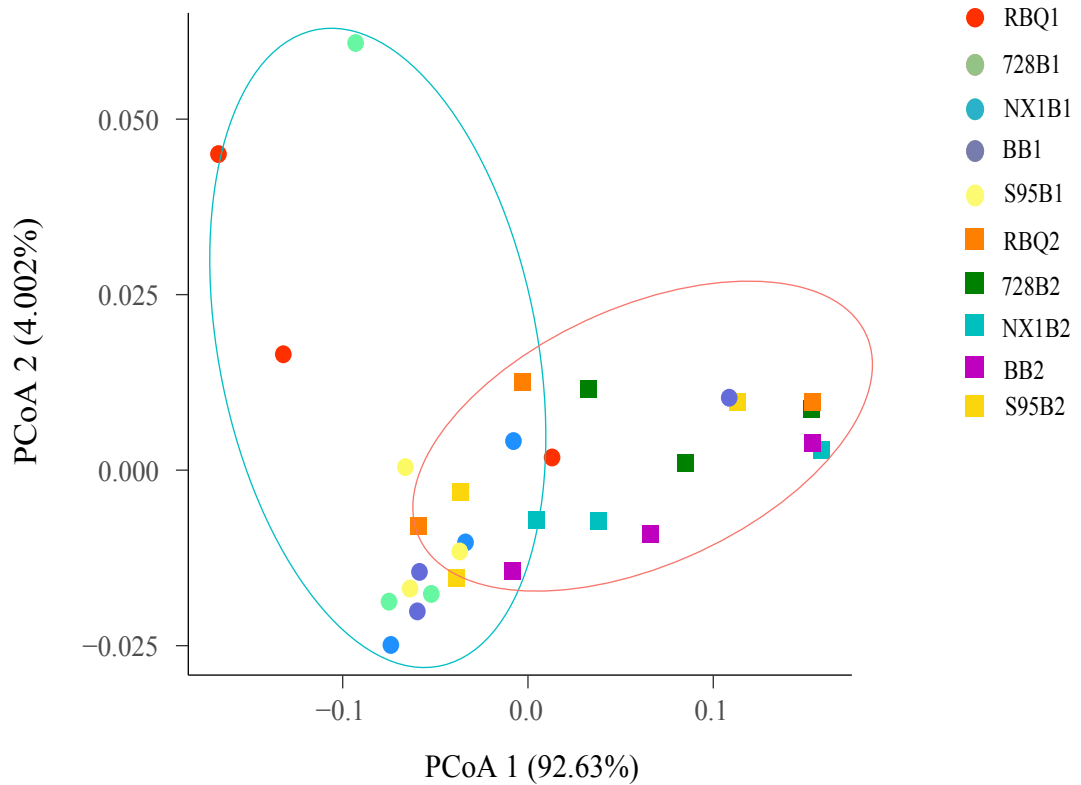

Supplementary Figure 3 Principal coordinates analysis (PCoA) based on the predicted functions.

Supplement: Supplementary file 3 [file Data_Sheet_3.PDF]
