# Supplementary material for: Unveiling Endophytic Bacterial Community Structures of Different Rice Cultivars Grown in a Cadmium-Contaminated Paddy Field
Source: Front Microbiol. 2021 Nov 16;12:756327. doi: 10.3389/fmicb.2021.756327 (PMC8635021; doi:10.3389/fmicb.2021.756327)
Supplement: Supplementary file 4 [file Data_Sheet_4.PDF]

Supplementary Material

Supplementary Tables

Supplementary Table 1 Comparison of α-diversity indices of bacterial communities between two developmental stages by using two-tailed t-test

Index	Vegetative stage<br>Mean ± SEM	Reproductive stage<br>Mean ± SEM	t	df	Sig.<br>（two-tailed）
Observed OTUs	369 ± 58	621 ± 58	-3.05	28	0.005**
Shannon	1.72 ± 0.29	3.06 ± 0.37	-2.86	28	0.008**
Simpson	0.54 ± 0.07	0.28 ± 0.07	2.63	28	0.014**
ACE	619.28 ± 77.51	996.41 ± 70.15	-3.61	28	0.001**
Chao	549.02 ± 81.01	901.54 ± 72.85	-3.24	28	0.003**

Supplementary Table 2 ANOVA analysis of α-diversity indices of bacterial communities among different cultivars

Sample*	Observed OTUs<br>Mean ±SEM	Shannon<br>Mean ±SEM	Simpson<br>Mean ±SEM	Ace<br>Mean ±SEM	Chao<br>Mean ±SEM
RBQ1	376 ± 191 a	2.19 ± 0.42 a	0.33 ± 0.04 a	626.78 ± 269.90 a	588.49 ± 284.62 a
728B1	309 ± 69 a	1.55 ± 0.80 a	0.59 ± 0.22 a	556.73 ± 42.22 a	456.91 ± 63.24 a
NX1B1	356 ± 163 a	1.54 ± 0.73 a	0.58 ± 0.18 a	682.32 ± 173.61 a	553.52 ± 214.52 a
BB1	448 ± 209 a	2.00 ± 1.16 a	0.52 ± 0.23 a	669.76 ± 299.77 a	630.34 ± 284.83 a
S95B1	359 ± 59 a	1.32 ± 0.23 a	0.66 ± 0.05 a	560.80 ± 111.48 a	515.84 ± 93.07 a
RBQ2	686 ± 181 a	3.23 ± 1.15 a	0.32 ± 0.18 a	1042.62 ± 145.63 a	975.36 ± 190.58 a
728B2	674 ± 77 a	3.59 ± 0.51 a	0.14 ± 0.05 a	1114.19 ± 141.76 a	1001.14 ± 126.20 a
NX1B2	691 ± 86 a	3.12 ± 0.79 a	0.27 ± 0.13 a	1103.44 ± 80.64 a	1003.39 ± 96.96 a
BB2	540 ± 190 a	2.85 ± 1.09 a	0.28 ± 0.19 a	915.51 ± 199.85 a	790.79 ± 217.63 a
S95B2	514 ± 145 a	2.51 ± 1.00 a	0.41 ± 0.20 a	806.27 ± 222.30 a	737.00 ± 210.24 a

* Cultivate name followed by 1 represents vegetative stage, 2 represents reproductive stage, hereafter.

Supplementary Table 3 Comparison of the class abundance of bacterial communities between two developmental stages by using two-tailed *t*-test

Class	Vegetative stage Mean ±SEM	Reproductive stage Mean ±SEM	t	df	Sig.（two-tailed）
Gammaproteobacteria	0.68 ±0.05	0.44 ±0.04	3.89	8.00	0.005**
Betaproteobacteria	0.08 ±0.02	0.20 ±0.03	-3.54	8.00	0.008**
Clostridia	0.11 ±0.05	0.05 ±0.01	1.31	4.46	0.252
Alphaproteobacteria	0.03 ±0.00	0.13 ±0.02	-6.61	4.38	0.002**
Actinobacteria	0.03 ±0.01	0.07 ±0.01	-2.52	8.00	0.036*
Deltaproteobacteria	0.02 ±0.01	0.05 ±0.01	-2.79	8.00	0.024*
Bacilli	0.02 ±0.00	0.01 ±0.00	1.16	5.11	0.297
Acidobacteria	0.01 ±0.01	0.01 ±0.00	-0.58	8.00	0.576

Supplementary Table 4 Comparison of the genus abundance of bacterial communities between two developmental stages by using two-tailed *t*-test

Genus	Vegetative stage Mean ±SEM	Reproductive stage Mean ±SEM	t	df	Sig.（two-tailed）
Pseudomonas	0.680 ±0.050	0.422 ±0.040	4.02	8.00	0.004**
Ralstonia	0.036 ±0.014	0.046 ±0.011	-0.54	8.00	0.602
Burkholderia-Paraburkholderia	0.013 ±0.005	0.065 ±0.013	-3.73	8.00	0.006**
Bradyrhizobium	0.008 ±0.003	0.039 ±0.009	-3.15	8.00	0.014**
*Clostridium_sensu_stricto*_11	0.045 ±0.039	0.001 ±0.000	1.12	4.00	0.326
*Clostridium_sensu_stricto*_1	0.017 ±0.010	0.011 ±0.004	0.56	8.00	0.593
Sideroxydans	0.003 ±0.001	0.024 ±0.002	-8.35	8.00	0.00**
Kineosporia	0.007 ±0.002	0.018 ±0.002	-3.86	8.00	0.005**
Anaeromyxobacter	0.013 ±0.005	0.012 ±0.002	0.22	8.00	0.833
unclassified_f__Rhodocyclaceae	0.005 ±0.001	0.018 ±0.001	-8.10	8.00	0.00**
Bacillus	0.013 ±0.003	0.009 ±0.002	1.22	5.98	0.268

Supplementary Table 5 Topological properties of co-occurrence networks of bacterial communities at two developmental stages

**Network parameters**	Vegetative stage	Reproductive stage
No. edges	4277	11659
No. pos. edges	4215	10763
No. neg. edges	62	896
No. vertices	266	425
Connectance	0.12	0.13
Average degree	32.16	54.87
Average path length	3.19	2.94
Diameter	9	12
Clustering.coefficient	0.749	0.61
No. clusters	6	2
Centralization degree	0.23	0.29
Centralization betweenness	0.06	0.07
Centralization closeness	0.02	0.08

Supplementary Table 6 Degree of connection for each class in bacterial community network at two developmental stages

Distribution of degree of connection	Vegetative stage			Reproductive stage		
	Tot* %	(-) %	(+) %	Tot %	(-) %	(+) %
Actinobacteria	17.4	0.5	99.5	9.2	9.4	90.6
Betaproteobacteria	16.8	2.7	97.3	23.9	6.9	93.1
Deltaproteobacteria	14.4	0.5	99.5	22.4	4.7	95.3
Alphaproteobacteria	11.3	0.8	99.2	7.1	3.4	96.6
Clostridia	9.8	3.9	96.1	3.8	56.6	43.4
Acidobacteria	6.4	0.6	99.5	6.4	3.3	96.7
Spirochaetes	5.2	1.1	98.9	4.8	3.6	96.4
Gammaproteobacteria	4.1	3.5	96.5	3.5	13.8	86.2
Bacilli	2.7	2.1	97.9	0.3	12.1	87.9
Bacteroidia	2.6	0.5	99.6	1.5	5.1	94.9
Ignavibacteria	1.7	1.4	98.6	3.3	5.1	94.9
Fibrobacteria	1.2	0	100	1.0	3.4	96.6
Unassigned	1.2	0	100	2.7	4.1	95.9
Nitrospira	1.1	0	100	4.5	3.6	96.5
VadinHA49	1.0	1.1	98.9	0	0	0
WCHB1-32	0.7	5.3	94.7	0.7	4.7	95.3
OPB35_soil_group	0.7	0	100	1.0	3.1	96.9
Negativicutes	0.6	0	100	0.1	59.3	40.7
SB-5	0.4	5.3	94.7	1.7	4.9	95.1
SJA-15	0.3	0	100	0.3	1.5	98.5
Bacoteroidetes_vadinHA17	0.3	0	100	0.8	4.5	95.5
Ktedonobacteria	0	0	0	0.1	3.1	96.9
Gemmatimonadetes	0	0	0	0.7	2.7	97.3
Flavobacteriia	0	0	0	0	66.7	33.3
Sphingobacteriia	0	0	0	0.2	5.5	94.6
others	0.2	0	100	0.2	0	100
Number of total degree of connection		8554			23318	

*Tot represents the percentage of each taxa connection in the total degree, + and - represent the percentage of co-occurrence and mutual exclusion, respectively.

Supplementary Table 7 Spearman correlation analysis of genus abundance with Cd content in roots

Phylum	Class	Family	Genus	Correlation Coefficient
Acidobacteria	*Acidobacteria*	*Holophagaceae*	*Geothrix*	0.879**
		Unknown_Family_o__Holophagae_Incertae_Sedis	*Thermoanaerobaculum*	0.768**
Actinobacteria	*Actinobacteria*	*Acidothermaceae*	*Acidothermus*	0.806**
		Micromonosporaceae	*Actinoplanes*	0.792**
			Micromonospora	0.830**
		Micrococcaceae	*Arthrobacter*	-0.830**
		Kineosporiaceae	*Kineococcus*	0.782**
			Kineosporia	0.794**
		Streptomycetaceae	*Streptacidiphilus*	-0.853**
Bacteroidetes	*Bacteroidia*	*Prolixibacteraceae*	*Prolixibacter*	-0.769**
		Porphyromonadaceae	*Microbacter*	0.880**
Firmicutes	*Clostridia*	*Family_XII_o__Clostridiales*	*Acidaminobacter*	0.772**
Proteobacteria	*Alphaproteobacteria*	*Hyphomicrobiaceae*	*Hyphomicrobium*	0.845**
		Rhizobiales_Incertae_Sedis	*Bauldia*	0.802**
		Roseiarcaceae	*Roseiarcus*	0.900**
		Xanthobacteraceae	*Pseudolabrys*	0.806**
			Variibacter	0.879**
	Betaproteobacteria	*Alcaligenaceae*	*Derxia*	0.824**
		Burkholderiaceae	*Chitinimonas*	0.889**
		Comamonadaceae	*Ideonella*	0.835**
		Gallionellaceae	*Candidatus_Nitrotoga*	0.824**
		Hydrogenophilaceae	*Ferritrophicum*	0.889**
			Thiobacillus	0.839**
		Neisseriaceae	*Chromobacterium*	0.881**
			Paludibacterium	0.819**
			Pseudogulbenkiania	0.799**
		Rhodocyclaceae	*Azospira*	0.774**
			Propionivibrio	0.846**
			Uliginosibacterium	0.879**
	Gammaproteobacteria	*Xanthomonadales_Incertae_Sedis*	*Acidibacter*	0.784**
		Xanthomonadaceae	*Dokdonella*	0.813**
		Pseudomonadaceae	*Pseudomonas*	-0.806**
		Coxiellaceae	*Coxiella*	0.869**
	Deltaproteobacteria	*Syntrophobacteraceae*	*Desulfovirga*	0.895**
		Syntrophaceae	*Desulfobacca*	0.779**
			Desulfomonile	0.875**
		Sandaracinaceae	*Sandaracinus*	0.775**
		Haliangiaceae	*Haliangium*	0.833**
		Geobacteraceae	*Geobacter*	0.891**
		Desulfovibrionaceae	*Desulfovibrio*	0.936**
		Desulfobulbaceae	*[Desulfobacterium]_catecholicum_group*	0.934**
			Desulfobulbus	0.834**
			Desulfocapsa	0.797**
Spirochaetes	*Spirochaetia*	*Leptospiraceae*	*Leptonema*	0.842**
		Spirochaetaceae	*Spirochaeta_2*	0.802**

Supplementary Table 8 The relative abundance of CSR traits at vegetative and reproductive stages

Functional traits	KEGG pathways	Vegetative stage	Reproductive stage	F	Sig.
		Mean ±SEM ($\times10^{-4}$)	Mean ±SEM (10^{-4})		
Competitive traits （C）	ABC transporters*	378.63 ±3.36	368.30 ±7.70	1.516	0.229
	**Biosynthesis of siderophore group nonribosomal peptides**	4.71 ±0.06	5.67 ±0.26	12.864	**0.001***
	Polyketide sugar unit biosynthesis	11.15 ±0.39	12.24 ±0.48	3.15	0.087
	Biosynthesis of type II polyketide backbone	0.14 ±0.09	0.04 ±0.01	1.255	0.272
	**Biosynthesis of type II polyketide products**	0.02 ±0.01	0.09 ±0.03	5.736	**0.024***
Stress tolerant traits （S）	Alanine, aspartate and glutamate metabolism	79.35 ±0.88	81.39 ±0.78	2.997	0.094
	**D-Glutamine and D-glutamate metabolism**	11.44 ±0.08	10.79 ±0.11	22.007	**0***
	Glycosphingolipid biosynthesis - globo series	0.85 ±0.22	1.40 ±0.21	3.455	0.074
	**Lipopolysaccharide biosynthesis proteins**	60.41 ±2.39	50.70 ±2.18	8.989	**0.006***
	Peptidoglycan biosynthesis	52.16 ±1.10	54.15 ±1.30	1.363	0.253
	ABC transporters*	378.63 ±3.36	368.30 ±7.70	1.516	0.229
	Ascorbate and aldarate metabolism	18.57 ±0.60	18.96 ±0.75	0.17	0.684
	Mismatch repair	51.84 ±1.10	51.98 ±1.12	0.008	0.93
	**Porphyrin and chlorophyll metabolism**	90.56 ±1.05	93.43 ±0.83	4.578	**0.041***
	**Proteasome**	2.88 ±0.14	3.34 ±0.17	4.299	**0.047***
	Thiamine metabolism	26.97 ±1.15	29.67 ±1.13	2.814	0.105
Ruderal traits （R）	**Carbon fixation pathways in prokaryotes**	92.84 ±1.56	99.62 ±2.22	6.238	**0.019***
	**Citrate cycle (TCA cycle)**	59.09 ±1.70	68.26 ±2.42	9.612	**0.004***
	**Oxidative phosphorylation**	119.54 ±1.95	129.17 ±3.24	6.506	**0.017***
	Ribosome	138.25 ±3.02	141.64 ±3.46	0.545	0.467
	Aminoacyl-tRNA biosynthesis	75.50 ±2.02	79.34 ±2.21	1.651	0.209
	Pyrimidine metabolism	108.29 ±2.50	109.28 ±2.07	0.092	0.764
	One carbon pool by folate	43.31 ±0.42	42.28 ±0.41	3.094	0.09

*Bold letters indicate metabolic pathways that change significantly during reproductive growth. The detailed criteria for functional traits was decribed by Wood et al. (2018).

Supplementary Table 9 Identity of the bacterial isolates based on 16S rDNA sequence similarity

Class	No.	Closest match species	Accession No.	Pairwise Similarity(%)
Bacilli	FS-3	*Bacillus albus*	MAOE01000087	99.79
	TB-1	*Bacillus aryabhattai*	EF114313	100.00
	TB-2	*Bacillus aryabhattai*	EF114313	99.72
	RB-1	*Bacillus aryabhattai*	EF114313	99.65
	TBB-1	*Bacillus aryabhattai*	EF114313	100.00
	TBB-2	*Bacillus aryabhattai*	EF114313	99.93
	TN-1	*Bacillus aryabhattai*	EF114313	100.00
	TN-2	*Bacillus aryabhattai*	EF114313	100.00
	RN-1	*Bacillus aryabhattai*	EF114313	99.65
	RS-2	*Bacillus aryabhattai*	EF114313	99.86
	FS-2	*Bacillus aryabhattai*	EF114313	99.65
	TB-3	*Bacillus cereus*	AE016877	100.00
	TS-1	*Bacillus cereus*	AE016877	99.86
	RS-1	*Bacillus cereus*	AE016877	99.86
	TB-4	*Bacillus koreensis*	LILC01000014	99.86
	RBB-2	*Bacillus siamensis*	AJVF01000043	99.79
	RN-2	*Bacillus siamensis*	AJVF01000043	99.44
	LS-1	*Bacillus toyonensis*	CP006863	99.79
	TBB-3	*Bacillus velezensis*	AY603658	99.64
	TN-3	*Bacillus velezensis*	AY603658	99.71
	TN-4	*Bacillus velezensis*	AY603658	99.78
	RN-3	*Bacillus velezensis*	AY603658	99.78
	RBB-1	*Bacillus zanthoxyli*	KX865140	99.79
	TS-2	*Paenibacillus cucumis*	KU201962	99.65
	FN-3	*Paenibacillus hunanensis*	EU741036	99.65
	LN-3	*Paenibacillus hunanensis*	EU741036	99.58
Alphaproteobacteria	LN-4	*Achromobacter ruhlandii*	CADIJL010000070	99.79
Betaproteobacteria	LBB-1	*Azospirillum palustre*	DQ787330	98.81
	2-N1	*Burkholderia vietnamiensis*	CP009631	1.00
	LB-1	*Herbaspirillum seropedicae*	CP011930	99.29
	FB-1	*Herbaspirillum seropedicae*	CP011930	99.57
	FS-4	*Herbaspirillum seropedicae*	CP011930	99.64
Gammaproteobacteria	RBB-3	*Klebsiella quasipneumoniae*	HG933296	99.50
	FN-2	*Klebsiella quasipneumoniae*	CBZR010000040	99.43
	LN-1	*Klebsiella quasipneumoniae*	CBZR010000040	99.43
	ABB-1	*Klebsiella quasivariicola*	CP022823	99.43
	FBB-1	*Klebsiella quasivariicola*	CP022823	99.36
	FN-1	*Citrobacter bitternis*	KJ817168	99.56
	LN-2	*Citrobacter bitternis*	KJ817168	99.56
	FS-1	*Luteibacter yeojuensis*	DQ181549	98.65
	LBB	*Pantoea dispersa*	DQ504305	100.00
	4-N2	*Pseudomonas sesami*	EU912472	0.99

Actinobacteria	LBB-2	*Microbacterium binotii*	EF567306	100.00

Supplementary Table 10 Cd-resistance (MIC) and PGP traits of bacterial isolates

No.	Top-hit taxon	MIC (μM)	IAA （μg/mL）	Siderophore （mg/L）	phosphate solubilization （mg/L）	ACC deaminase （U/mg）
TB1	*Bacillus aryabhattai*	40	17.92 ±0.26	30.77 ±0.66	8.50 ±0.13	12.84 ±0.70
TN1	*Bacillus aryabhattai*	40	20.32 ±0.15	26.22 ±0.15	8.77 ±0.33	1.12 ±0.19
TBB1	*Bacillus aryabhattai*	60	5.98 ±0.38	40.87 ±9.85	11.25 ±8.73	none
FS-2	*Bacillus aryabhattai*	20	27.15±0.03	11.53±0.10	0.58±0.03	none
RS2	*Bacillus aryabhattai*	40	20.19 ±1.54	11.33 ±0.81	23.17 ±1.94	6.26 ±0.64
TB3	*Bacillus cereus*	1280	1.43 ±0.14	32.59 ±0.26	39.23 ±1.59	1.45 ±0.07
TS1	*Bacillus cereus*	1280	0.90 ±0.14	48.49 ±3.13	14.58 ±9.26	1.39 ±0.26
TB4	*Bacillus koreensis*	60	7.52 ±0.43	27.74 ±8.34	13.27 ±0.79	1.76 ±0.55
TN3	*Bacillus velezensis*	80	2.92 ±0.38	24.06 ±3.54	7.05 ±0.84	none
TBB3	*Bacillus velezensis*	80	3.52 ±0.09	7.89 ±4.04	5.86 ±3.83	0.22 ±0.12
RN2	*Bacillus siamensis*	80	3.59 ±0.01	18.39 ±1.92	10.35 ±0.51	3.67 ±1.07
RBB2	*Bacillus siamensis*	80	1.78 ±0.02	25.77 ±10.91	7.01 ±3.03	1.2 ±0.26
RBB1	*Bacillus zanthoxyli*	10	17.31 ±1.09	13.59 ±0.35	14.11 ±0.75	none
TS2	*Paenibacillus cucumis*	80	1.45 ±0.21	none	31.00 ±1.62	0.51 ±0.02
LN-4	*Achromobacter ruhlandii*	640	none	13.75 ±0.20	0.32 ±0.02	0.38 ±0.03
LBB-1	*Azospirillum palustre*	40	9.57 ±0.94	14.00 ±0.35	none	0.49 ±0.03
2-N1	*Burkholderia vietnamiensis*	320	42.77 ±0.80	27.61 ±4.93	24.60 ±0.57	0.92 ±0.01
LB-1	*Herbaspirillum seropedicae*	20	15.57 ±0.10	13.49 ±0.05	0.18 ±0.02	0.39 ±0.05
RBB3	*Klebsiella quasipneumoniae*	640	52.84 ±7.00	19.61 ±1.22	33.70 ±4.32	5.38 ±0.25
FN-1	*Citrobacter bitternis*	320	27.17±0.05	5.92 ±0.86	none	1.31 ±0.003
LBB-3	*Pantoea dispersa*	320	8.93 ±0.13	8.04 ±0.15	0.21 ±0.05	0.43 ±0.02
FS-1	*Luteibacter yeojuensis*	160	0.28 ±0.06	14.10 ±0.15	0.45 ±0.05	4.89 ±0.96
4-N2	*Pseudomonas sesami*	1280	4.91 ±0.38	96.85 ±1.85	41.46 ±1.16	2.32 ±0.19
LBB-2	*Microbacterium binotii*	160	1.01 ±0.16	0.87 ±0.15	1.54 ±0.33	1.38 ±0.15

*
